# OxInflammation: From Subclinical Condition to Pathological Biomarker

**DOI:** 10.3389/fphys.2018.00858

**Published:** 2018-07-09

**Authors:** Giuseppe Valacchi, Fabio Virgili, Carlo Cervellati, Alessandra Pecorelli

**Affiliations:** ^1^Plants for Human Health Institute, Department of Animal Sciences, North Carolina State University, Kannapolis, NC, United States; ^2^Department of Life Sciences and Biotechnology, University of Ferrara, Ferrara, Italy; ^3^Council for Agricultural Research and Economics - Food and Nutrition Research Centre (C.R.E.A.-AN), Rome, Italy; ^4^Department of Biomedical and Specialist Surgical Sciences, University of Ferrara, Ferrara, Italy

**Keywords:** biomarkers, neurological disorders, oxidative stress, inflammation mediators, NFκB

## Abstract

Inflammation is a complex systemic response evolved to cope with cellular injury, either due to infectious agents or, in general, with sporadic events challenging tissue integrity and function. Researchers involved in different fields have the tendency to look at the inflammatory response with different angles, according to their specific interest. Established its complexity, one of the most evident features of the inflammatory response is the generation of a pro-oxidative environment due to the production of high fluxes of pro-oxidant species. This production begins locally, close to the sites of tissue damage or infection, but eventually becomes a chronic challenge for the organism, if the inflammatory response is not properly controlled. In this review, we focus on this specific aspect of chronic, low-level sub-clinical inflammatory response. We propose the term “OxInflammation” as a novel operative term describing a permanent pro-oxidative feature that interact, in a positive feed-back manner, to a not yet clinically detectable inflammatory process, leading in a long run (chronically) to a systemic/local damage, as a consequence of the cross talk between inflammatory, and oxidative stress mediators. Therefore, it could be useful to analyze inflammatory markers in pathologies where there is an alteration of the redox homeostasis, although an inflammatory status is not clinically evident.

## Inflammation as a common feature of dysfunction and disease

Inflammation is a tissue response to damage characterized by a fine regulated set of events in which several cell type are sequentially activated and able to secrete key mediators (Calder et al., 2013).

With the term inflammation it is usually indicated a confined response to injury or infection characterized by phenomena such as: increased blood flow, capillary dilatation, leucocyte inflltration, and the release of chemical mediators, involved in initiating the damaged tissue repair, and the elimination of noxious agents (Calder et al., 2013).

The main cells involved in the inflammatory responses are neutrophils, macrophages, and other immune cells, controlled by chemical mediators generically called cytokines and chemokines (Mantovani et al., 2011). Indeed, the first step of an inflammatory response is the up-regulation of genes encoding for cytokines, chemokines, and other mediators, through activation of transcription factors such as nuclear factor κ B (NF-κB), activator protein-1 (AP-1), nuclear factor of activated T cells (NFAT), and signal transducer and activator of transcription-3 (STAT3) (Smale, 2010). The activation of the inflammasome, a multiprotein complex, that serves as a platform for caspase-1 activation and resulting in the proteolytic maturation and secretion of interleukin (IL)-1β and IL-18, is another alternative pathway triggering an inflammatory response (Guo et al., 2015).

The production of specific cytokines or chemokines immediately results in leukocytes recruitment to the site of damage or infection, where they express adhesion properties and produce bactericidal chemicals (including reactive oxygen and nitrogen species, ROS and RNS) and further inflammatory mediators to increase local blood flow and activate phagocytic cells, including macrophages, to eliminate dead cells and tissue debris.

In general, the acute inflammatory episodes are terminated by the removal of pathogens and exogenous agents, (resolution of inflammation), that lead to the inflammatory cells clearance. Several mechanisms work in a coordinated way to consent the proper inflammatory resolution. This very fine and coordinated process aimed to re-establish tissue integrity and physiology. Overall, the block of chemokine signal prevents further neutrophil infiltration, while immune cells such as monocytes and macrophages are attracted by apoptotic neutrophils. Then, the uptake of apoptotic neutrophils by macrophages switch those cells to the resolving phenotype, crucial event to restore tissue homeostasis. Successively, endothelial cells and fibroblasts are then involved tissue repair thanks also to the anti-inflammatory molecules involved in the feedback mechanism responsible for the “switches off” the inflammatory event (Serhan and Savill, 2005). In other words, inflammation is (or should be) a rapid, intense response aimed to tissue protection, and recovery, promptly controlled to avoid possible detrimental consequences arising from an “over-response.”

Very often two distinct types of inflammation are considered: the acute and the chronic inflammatory response, according not only with the length of occurrence, but also with the intensity of the cellular response, low grade and high grade, respectively. In general, when the tissue offense is removed, the infection has been cleared, and the damaged tissue repaired we have the so called “inflammation resolution.”

As mentioned above, the inflammation is switched off when feedback mechanisms trigger the expression of anti-inflammatory molecules, inducing lipoxins, bioactive autacoid metabolites of arachidonic acid that modulate the transition from neutrophil to monocyte recruitment, the clearance of cells debris and the beginning of tissue repair at the related site (Serhan and Savill, 2005). In some cases, when the trigger(s) that activated the acute inflammatory response persist or, for different causes, the resolution phase is not efficient, a chronic inflammatory state may occur.

Typically, this condition takes place in the presence of chronic infections, unrepaired tissue damage, persistent allergens, but also in the presence of foreign particles, such as environmental nanoparticles or cigarette smoking (Nathan and Ding, 2010; Magnani et al., 2016). In these cases, chronic inflammation is localized and somehow confined to the site of original inflammatory inducer. This condition often results in different types of local tissue remodeling, such as the formation of granulomas and the generation of tertiary lymphoid organs at the site of infection. In the airways, persistent chronic inflammation induced by allergens or pollutant nanoparticles associates to lung epithelial tissue remodeling, inducing in turn asthma and respiratory dysfunction (Murdoch and Lloyd, 2010).

In the recent years, a growing number of chronic inflammatory conditions have been recognized where the initiating trigger is not well defined but does not seem to involve infection or tissue damage. These inflammatory conditions are of particular interest because they accompany many diseases of industrialized countries, including obesity, and type 2 diabetes, atherosclerosis, neurodegenerative diseases, cancer, and aging (Monti et al., 2016).

## Inflammation in hereditable diseases

Under this perspective, chronic inflammation plays an important role in determining a vicious cycle with the associated pathological process, either associated with inherited genetic diseases. For instance, children with the trisomy 21 have a differential expression of genes related to inflammation (BDKRB1 and LTA4H) not located on chromosome 21 and higher levels of pro-inflammatory cytokines compared with normal children, contributing to the systemic aspects of the disease (Broers et al., 2012). In adults with Down's syndrome, the immunophenotypic alterations have been attributed to an early onset of immunosenescence associated to a dysregulation of NF-κB transcription factor (Kim et al., 2006; Trotta et al., 2011).

Similarly, the Rett syndrome (RTT), an orphan progressive neurodevelopmental disorder due to a mutation in the methyl-CpG binding protein 2 gene (*MECP2*), is characterized by an altered cytokine profile. More specifically, T helper Type 2-(Th2) cytokine response, and TH2 cytokines IL-9, and IL-13 levels have been found up regulated in peripheral blood mononuclear cells (PBMC) of RTT patients compared to controls (Pecorelli et al., 2016a). In addition, Sickle cell disease, a monogenic globin disorder characterized by the production of structurally abnormal hemoglobin, is associated with a dysregulation of the inflammatory response resulting in chronic inflammation. Systemic inflammation is evident in Sickle cell patients that present elevated steady-state concentrations of several plasma inflammatory markers, such as IL-1, IL-6, and IL-8, and TNF-α, which are elevated further during acute episodes of illness (Owusu-Ansah et al., 2016).

## Inflammation in non-hereditable metabolic diseases

Beside “genetic disease,” also other degenerative diseases due to the combination of genetic risk and life style factors such as the obesity and obesity-associated metabolic syndrome have a significant inflammatory component. For instance, there is solid evidence that obesity is associated with inflammation, and that chronic inflammation can promote obesity-associated diabetes, in part by inducing insulin resistance (Hotamisligil, 2006). Similar positive feedback loops are present in atherosclerosis, obesity, cancer, and other chronic inflammatory diseases (Pawelec et al., 2014; Kolb et al., 2016; Soeki and Sata, 2016). Indeed, this type of reciprocal relationship may be responsible, at least in part, for the chronic nature of these inflammatory conditions and distinguishes them from the first type of chronic inflammation, which is caused by persistence of the inflammatory inducer, e.g., a microbial/viral invasion or a physical injury.

Obesity is an emblematic example of a negative loop between a pathological (or pre-pathological) phenotype and chronic inflammation. Actually, inflammation has been proposed to be one a major factor involved in the progression of the large spectrum of dysfunctions, finally resulting in an overt disease associated to a positive energy balance, overweight, and eventually to obesity (Hotamisligil, 2006). Obesity-associated inflammation is first triggered by excess nutrients that allows to activate metabolic signaling pathways such as c-Jun N-terminal kinase (JNK), NF-κB, and protein kinase R (PKR) (Solinas and Karin, 2010). Activation of these pathways leads to the release of inflammatory cytokines resulting in a low-grade inflammatory response (Gregor and Hotamisligil, 2011). In addition, obesity is also linked to the increased endoplasmic reticulum stress and the activation of unfolded protein machinery, able to activate NF-κB, JNK, and induce a redox perturbation defined oxidative stress, that will further up-regulate inflammatory cytokines release (Maher Cnop et al., 2012). Altogether, these pathways initiate and sustain obesity-associated inflammation, which is initially confined in white adipose tissue, but eventually spread to other tissues including the liver, pancreas, and brain (Cildir et al., 2013). This condition determines a “systemic low-grade inflammatory response” that affect immune cell infiltration and polarization toward adipose tissue (Hotamisligil, 2006). Indeed, macrophages are recruited in the white adipose tissue in obesity condition, being the main source of cytokines release in this tissue (Weisberg et al., 2003).

Overweight and obesity are major drivers of a complex and undefined pre-pathological condition referred as “metabolic syndrome.” In fact, several pathologies such as type 2 diabetes, atherosclerosis and cardiovascular diseases are all often a consequence of the development of metabolic syndrome which is characterized by high blood pressure, atherogenic dyslipidemia, insulin resistance, and increased glucose levels, a pro-thrombotic state and, according to the matter of this commentary, to a mild, persistent pro-inflammatory state (Esser et al., 2014).

## Oxidative stress: a failed physiological response to a challenge

Living in an aerobic environment, the generation of potentially noxious oxidants is, of course, an inevitable phenomenon. Ever since oxygen became an important component of the atmosphere, aerobic organisms evolved to utilize it for a much better energetic exploitation of organic substrates and also evolved strategies to embody oxygen and oxygen-derived molecules in intracellular signaling and within defense strategies from potentially harmful events.

In order to get to this goal, efficient machineries had to evolve too, able to overcome the consequences of an “oxidative challenge,” due to the presence of oxygen or oxygen derived reactive species (Maher and Yamamoto, 2010). The consequences of oxidative challenges are frequently referred as “oxidative stress,” which is probably one of the less defined and abused term in biochemistry and cellular physiology. According to one of the most comprehensive and acceptable definition, oxidative stress can be considered “the consequence of the failure to maintain the physiological redox steady state, which is the self-correcting physiological response to different challenges” (Ursini et al., 2016). The important and “dual” role of oxygen and ROS in aerobic organism implies that a moderate and finely tuned production of reactive species is crucial in cell biology within a certain range and the possibly excess of ROS is physiologically prevented by a cellular specific defensive system. The perturbation of this physiological adaptive dynamic equilibrium, induced by a persistent challenge or inappropriate feedback response, leads to the inflammatory state (Forman et al., 2014; Ursini et al., 2016).

## Main players in redox homeostasis

Thus, ROS and RNS, main cell electrophiles, are involved in an extremely delicate and easily corruptible balance between harm (i.e., biological damage) and benefit (i.e., redox signaling). The need of a perpetual flux of these oxidants is primarily satisfied by mitochondria, since the physiological electron leakage from respiratory chain (complex I, II, and III) results in a generation of anion superoxide (Rimessi et al., 2016). Interestingly, the extent of mitochondria contribution in cellular ROS has been recently questioned (Fridovich, 2004), on the basis of novel findings suggesting that this source is much less quantitatively relevant in respect to the original estimation by Chance et al. (1979). Remaining in mitochondrial environment, there are other enzymes capable to participate in ROS production, such as dihydrolipamide dehydrogenase, monoamine oxidase etc. (Rimessi et al., 2016).

To preserve within a physiologically tolerable range the level of superoxide, mitochondria has been geared with the most efficient ROS scavenger, the manganese superoxide dismutase (MnSOD) which, as the other cytosolic isoenzyme (i.e., copper zinc SOD), is the only biological agent able to convert superoxide into hydrogen peroxide (Zou et al., 2017). Owing its relatively mild reactivity hydrogen peroxide can serve as the prominent component of intracellular signal transduction. However, when the concentration of this ROS exceeds the homeostatic level, it can give rise, especially in presence of free copper or iron, to the most potent free radical, hydroxyl radical, i.e., the main responsible of oxidative stress-related damage to proteins, lipids and DNA. One of the few reactive species with a comparable cytotoxic potentials is peroxynitrite, which stems from the combination between nitrogen monoxide and superoxide ion (Pacher et al., 2007). Glutathione peroxidase (Gpx) and catalase (CAT) play a prominent role in preventing from hydroxyl radical formation, because they can definitely covert hydrogen peroxide into water, although via different catalytic mechanisms. Gpx is greatly important in maintaining within a suitable concentration range the most abundant redox couple in a cell, reduced, and oxidized glutathione (GSH and GSSG, respectively) (Cadenas and Davies, 2000). The other enzymatic and non-enzymatic factors involved in glutathione cycle and other defensive agents (e.g., thioredoxin) have been already described in-depth in some fascinating previous reviews (Chance et al., 1979; Cadenas and Davies, 2000; Fridovich, 2004; Pacher et al., 2007; Forman et al., 2014; Rimessi et al., 2016; Ursini et al., 2016; Zou et al., 2017).

Besides mitochondria, there are other cellular constituents that directly participate in redox processes; for example, peroxisomes are rich in CAT, allowing them to cope with the elevated propensity of these organelles for the generation of superoxide anion and hydrogen peroxide (Davies et al., 2017). The endoplasmic reticulum also contributes to cellular levels of ROS, derived by protein folding processes and membrane bound NADPH oxidase (NOX) enzymes. Different location but similar catalysis products characterize other pro-oxidant enzymes such as lipoxygenases, cyclooxygenases, and xanthine oxidase (Cho et al., 2011).

It is undoubted that the complex network of defensive agents, including SOD, CAT, Gpx etc. acting, essentially, to prevent the formation of the highly cytotoxic hydroxyl radical (^.^OH), is the primary, in terms of both time, and importance, defensive layer against ROS/RNS induced damage. Possible leaks from this outpost can be then countered by further repair systems such as proteinases, lipases, DNA repair enzymes (Davies et al., 2017). Important defensive contribution is also afforded by proteasome, the ubiquitous multicatalytic protease responsible for degradation of intracellular target proteins, including oxidized proteins (Aiken et al., 2011). The proteasome is composed by a 20S particle harboring the proteolytic active sites, which is capped to one or both sides by the 19S regulatory particles, which assist the processing of poly-ubiquitinated substrates. The 20S exists also in the uncapped form which is proposed to cope with the degradation of oxidized and unfolded/aggregating-prone substrates (Schmidt and Finley, 2014). It has been shown that an increase in oxidant species leads to disassembly of 26S, into the catalytic cores 20S and the regulatory caps 19S. 20S, but not the 26S, is able to remove oxidized proteins thereby preventing their aggregation and cross-linking (Aiken et al., 2011; Demasi et al., 2014). Interestingly, it has been hypothesized that the effect of proteasome cell redox balance perturbation is biphasic: low levels of reactive species induces activation of 20S activity while high levels (and thus full-blown oxidative stress) an inhibition of its activity (mostly by posttranslational modification) (Aiken et al., 2011; Demasi et al., 2014).

## Mutual activation between inflammatory and oxidative stress mediators

Exogenous stressors (pollution, smoking, fat diet, etc.) or endogen factors (diseases and inflammation itself) are potentially able to disrupt this finely tuned homeostasis, inducing the formation of oxidants, so called electrophiles, such as ROS and RNS, that take one or two electrons from a nucleophile, from various sources (Cadenas and Davies, 2000; Forman et al., 2014).

Inflammation is indeed an important source of oxidative stress. In the course of an inflammatory event, oxidants molecules can originate from the activity of lipoxygenases, cyclooxygenases, xanthine oxidase, phagocytic, and non-phagocytic nicotinamide adenine dinucleotide phosphate oxidases (NOXs) and also from Fenton/Haber—Weiss reactions catalyzed by transition metals, possibly locally freed in the milieu of a physical injury and eventually released systemically (Forman and Torres, 2002; Bergamini et al., 2004; Valko et al., 2005; Sies, 2015). In particular, NOX2 and mitochondria-derived ROS are required for respiratory burst occurring in activated leukocyte. During this semi-physiological event, the levels of superoxide increase from picomolar to micromolar concentrations inside the cells within a few minutes (Sethi and Tabel, 1990; Wenzel et al., 2017). This makes systemic acute (e.g., sepsis), but also low-grade inflammation, a terrific source of ROS, as demonstrated by the several epidemiological studies reporting a strong and positive association between peripheral makers of inflammation and oxidative stress in individuals affected by various pathologies (Nonaka-Sarukawa et al., 2003; Abramson et al., 2005; Bougoulia et al., 2006; Cottone et al., 2006).

NOXs function and regulation are a clear-cut example of the mutual connection between redox processes and inflammation. Notably, the members of this membrane-bound multi-enzyme complex are the only endogenous source of ROS with the specific function to produce signaling free radicals molecules whereas all other aforementioned sources produce ROS either upon redox modifications (e.g., thiol oxidation in xanthine dehydrogenase generates the oxidase form) or by mitochondria (Wenzel et al., 2017). ROS (O_2_ and H_2_O_2_) generated by NOX_S_ essentially serve for microbial killing at sites of inflammation (NOX2) and redox signaling. Pro-inflammatory mediators, in primis TNFα and IL-1β, represent the major inducers of activation of both phagocytic and non-phagocytic NOX (Lee and Yang, 2012). In turn, the increase in ROS production can activate cells of immune system (but also of endothelium, and different types of epithelium) to induce protein kinase cascade (PKC, MAPKs etc.) and redox-sensitive transcription factors, in primis AP-1, and NF-kB. This concatenation of events ultimately brings about the expression of a number of pro-inflammatory mediators, including those involved in the stimulation of NOX. It is this ability to translate an inflammatory stimulus into pro-oxidant impulse that accounts for the widely postulated central role of NOXs in the pathogenesis of various diseases, such as cardiovascular diseases, lung diseases, cancer and Alzheimer's disease (AD) (Yang et al., 2007; Grammas, 2011; Lee and Yang, 2012; Heneka et al., 2015; Dias et al., 2016; Wenzel et al., 2017).

In conclusion, not only inflammatory mediators such as the release of cytokines are able to implement the production of ROS via the activation of specific enzyme present in the cells (NOX, XO, etc.) but also ROS can themselves modulate inflammation (Mittal et al., 2014). For instance, it has been well demonstrated that ROS can activate transcription factors involved in the inflammatory process such as NFAT-1, AP-1, HIF-1a, and NF-κB (Morgan and Liu, 2011). In addition, ROS can act as second messengers and induce a cascade of events by MAPK activation (p38, JNK, and ERK1/2) which can also lead to migration of inflammatory cells and therefore augment the inflammatory response (Son et al., 2013; Mittal et al., 2014). It is worth mentioning that, based on the ROS concentration, it is possible to have opposite results. Several authors have shown that an acute and intense oxidative stress can lead to the oxidation of NF-κB, affecting its ability to translocate to the nucleus and bind to the DNA (Morgan and Liu, 2011).

## Oxinflammation as specific feature in the pathogenesis of common and rare diseases

The central role of the reciprocal interplay between oxidative stress and inflammation is a general notion. In this review we want to point out that a specific chronic failure in the control of oxidative events, resulting in a mild, long-term pro-oxidative cellular environment, is a specific feature in the genesis of diseases.

As mentioned above, there are abundant lines of evidence indicating that a low grade/chronic inflammatory response, escaping for different reason to a resolving feedback regulation, and the presence of reactive species in the inflammatory processes are hallmark feature of several pathologies, ranging from genetically determined diseases, such as Rett, and Down syndrome, to the most common metabolic dysfunctions, including diabetes, cardiovascular disease, cancer, and neurodegenerative disorders, such as AD and Parkinson's disease (Kaneto et al., 2007; Pou et al., 2007; Yang et al., 2007; Perluigi and Butterfield, 2012; Heneka et al., 2015; Cervellati et al., 2016; Dias et al., 2016; Pecorelli et al., 2016b; Valacchi et al., 2017; Wenzel et al., 2017).

## Diabetes

Oxidative stress and inflammation are considered critical factors for the pathogenesis of diabetes mellitus (DM) type I and II and both plays a crucial role in the development of the frequent micro vascular and cardiovascular complications of this chronic disease (Kaneto et al., 2007). The most direct body evidence in favor of this biological link has been generated from the several population-based studies showing association between DM and systemic levels of inflammation or oxidative stress (Pradhana et al., 2001; Hu et al., 2004; Wu et al., 2004; Dalle-Donne et al., 2006). At least partially, the mechanistic explanation of how the mediators of redox/inflammation could act as downstream/upstream player in the impairment of endocrine activity of pancreas, insulin resistance, and diabetes-specific pathology have been illustrated in recent reviews (Baynes and Thorpe, 1999; Ceriello and Motz, 2004).

Type I DM is caused by the autoimmune destruction, with collateral and consequent inflammatory process of β cells of the endocrine pancreas. ROS/RNS, along with pro-inflammatory cytokines generated by islet-infiltrating immune cells, may contribute to the impairment of β-cell function, targeting cell metabolism and potassium (adenosine-5'-triphos-phate) channels (Drews et al., 2010) and inducing mitochondrial dysfunction (Rachek et al., 2006) [further worsened by hyperglycemia (Rolo and Palmeira, 2006)], thus exacerbating oxidative challenge against islet cells.

The implication of Oxidative stress and inflammation-related phenomena have been mostly studied in type II DM.

Growing lines of evidence suggest that oxidative stress may be the primary triggers of tissue damage due to hyperglycemia by several mechanisms. As recently described by Giacco et al. (Giacco and Brownlee, 2010) and others (Rolo and Palmeira, 2006), the proposed mechanisms consist in alteration of polyol and hexosamine pathway, increase in intracellular formation of advanced glycation end-products (AGEs) and their receptors (RAGEs), and activation of protein kinase C (PKC) isoforms.

One of the best known processes consists in the enhanced intracellular formation of advanced glycation end-products (AGEs), resulted from the non-enzymatic reaction of glucose and other reactive sugars with amino groups of amino acids (as well as lipid and DNA). This multi-step reaction is accelerated by ROS (Ahmed, 2005).

It has been suggested that the interaction with RAGEs might result in an increased production of reactive species (Giacco and Brownlee, 2010) (from mitochondria, auto-oxidation of glucose, and NOXs) and activation of various redox sensitive transcription factors such as NF-κB (Haslbeck et al., 2005), with subsequent upregulation of pro-inflammatory and thrombogenic genes. By fueling this pathways, the dichotomy AGE/RAGE might contribute to the development of typical diabetes complications, including polyneuropathy, retinopathy, and atherosclerosis (Haslbeck et al., 2005; Giacco and Brownlee, 2010). In support with this claim, transgenic mice lacking RAGE showed, less propensity to develop atheroma, possible effect of decreased expression of proinflammatory mediators, and ROS (Soro-Paavonen et al., 2008). Noteworthy, the vicious cycle involving oxidative stress and inflammation in diabetes find confirmation in the recent evidence that some pro-inflammatory agents might be better ligand for RAGEs than AGEs their self (Giacco and Brownlee, 2010).

Insulin resistance occurs in most of patients with type II DM, and this adverse metabolic condition is closely associated with overall and, mostly, central obesity (Esser et al., 2014). As already underpinned, obesity can be almost regarded as a synonymous of (systemic) inflammation (Gregor and Hotamisligil, 2011), although infrequent cases of metabolically healthy obesity exist. Elevated levels of the typical markers of obesity-induced low grade inflammation, hs-CRP and TNF-α are strongly predictors of type 2 DM in adults (Dandona et al., 2004). In turn, increased of these and other downstream- or upstream- linked cytokines promote enzymatic (in primis NOX) and non-enzymatic pro-oxidative processes.

## Cardiovascular disease

As discussed earlier, DM is a major risk factor for atherosclerosis, the leading cause of CVD. Like the disease of carbohydrate metabolism, atherosclerosis is widely referred as to chronic inflammatory pathology, where oxidative stress plays a crucial pathogenic role. Vascular sources of ROS/RNS are multiple and include mitochondria, the uncoupling nitric oxide synthase, and various enzymes, in particular xanthine oxidase, lipoxygenase, and NOX (Manea et al., 2015). The function of NOX as a “bridge” between inflammation and oxidative stress is essential in the development and progression of atherosclerosis. Various NOXs are constitutively expressed in endothelial cells, smooth muscle cells, adventitial fibroblasts, and circulating and tissue-resident immune cells participating in atherosclerotic processes [comprehensive review on the topic here (Manea et al., 2015; Kattoor et al., 2017)]. NOX isoforms involved in vascular pathologies appear to affect the activation of transcription factors (NF-kB, AP-1, and signal transducer and activator of transcription, STAT) found within atherosclerotic lesions and in the vascular wall of animals and human diabetic and hypertensive patients (Manea et al., 2015). It is well known that these factors are master regulators of genes associated with differentiation, proliferation, and migration of immune cells and resident vascular cells, and modulate the expression of a plethora of pro-inflammatory and immune factors (Celada et al., 1996). Relevant to the concept of OxInflammation as a self-perpetuating vicious cycle of cytotoxic substances coming from different sources, these redox-sensitive transcription factors, along with others involved in vascular remodeling, have been implicated in the regulation of vascular NOX (Madamanchi et al., 2005; Singh and Jialal, 2006). This regulation can occur directly, through direct transcription factor- NOX gene promoter interaction mechanisms, and/or indirectly, by upregulating the expression of pro-inflammatory cytokines which are agonist of NOX activity (Manea et al., 2015). The resulting, still not completely deciphered, redundant signaling pathways have been deemed to be intimately implicated in atherosclerosis onset.

Immune cells play a crucial role in the initiation, propagation, and both acute and chronic complications of CVD. Activated leukocytes participate in vascular inflammation response and secrete the content of their azurophilic granules in close vicinity to inflamed tissues (Huang et al., 2013). One of the main component of these granules is the heme protein Myeloperoxidase (MPO) (Huang et al., 2013). This enzyme, which is abundant in human atheroma and in patients affected by CVD (Podrez et al., 2000), is able to amplify the oxidative potential of its co-substrate H_2_O_2_ (derived from NOXs, xanthine oxidase etc.) forming potent oxidants (e.g., hypochlorous acid) capable of chlorinating and nitrating phenolic compounds (Carr et al., 2000). It has been shown that MPO, released within both the circulation and within atherosclerotic plaque, binds to high density lipoprotein (HDL), leading to oxidative modification and inactivation of proteins responsible of the antioxidant and anti-inflammatory proprieties of the lipoprotein (Huang et al., 2013). The major targets of MPO deleterious effect are the functionally couple of proteins, apoliprotein A1, and paraoxonase-1 (PON-1) (Cervellati et al., 2015a).

It has been suggested that the most important physiological function of PON1 is to contrast the oxidation of low density lipoproteins (LDLs) (Huang et al., 2013; Cervellati et al., 2018). This process, yielding to the formation of the highly pro-atherogenic oxidized LDL (oxLDL), is caused by the altered local and systemic production of ROS (Madamanchi et al., 2005). Ox-LDLs induce endothelial cell activation, dysfunction, death, and contribute causally to atherosclerosis, onset and progression, through (again) the activation of NF-κB and AP-1 pathways (Valente et al., 2014). Endothelial injury induces the expression of adhesion molecules and chemiotactic cytokines, thereby promoting activation and migration of immune cells vascular smooth muscle cell (Madamanchi et al., 2005). Subsequently, oxLDLs (but also not-modified LDLs) are transported into and across the endothelium, likely at the site of endothelial damage provoked by oxLDLs their-self (Madamanchi et al., 2005; Kraehling et al., 2016). Within the intima layer, these particles are further oxidized by ROS produced by resident macrophages, that take up oxLDL and become foam cells. These deadly cells exacerbates the inflammatory processes that terminate with atheroma formation (Madamanchi et al., 2005). Therefore, also oxLDL represent a player able to translate the oxidative challenge in an inflammatory event, which in turn can induce endogenous ROS formation and further feed the OxInflammation vicious cycle.

Both central and peripheral redox homeostasis dysregulation seem to occur in a very large spectrum of pathologies, if not all, regardless of the pronounced systemic involvement that characterizes any specific disease. Some tissues and organs are known be highly vulnerable to reactive species challenge. For instance, cells composing the brain have a long life, relatively low levels of endogenous antioxidants (particularly glutathione), high levels of peroxidizable polyunsaturated fatty acids, and high levels of pro-oxidant metals. Moreover, the high ratio of oxygen consumption further supports the “normal” physiological extent of ROS leakage from mitochondrial activity (Golden et al., 2002; Cervellati et al., 2014a, 2016). This organ-specific susceptibility to oxidative insults accounts, at least partially, to the well-known involvement of oxidative stress in several neurological pathologies such as Down's syndrome, AD, Parkinson's, etc. (Abramov et al., 2004; Olivieri et al., 2011; Cervellati et al., 2013, 2014a, 2016; Thanan et al., 2014). In the next session, we will mainly focus on the involvement of OxInflammation in brain related diseases.

## Alzheimer's disease

The “amyloid cascade hypothesis” is currently the most widely accepted model explaining AD aetiopathogenesis. β-amyloid (Aβ) deposition and aggregation would be the main and the first neuropathological event leading to the formation of senile plaques and then favoring the neurofibrillary tangles (NFT) formation (the other AD neuropathological hallmark), neuronal cell death, and dementia (Hardy and Higgins, 1992). Nevertheless, important concerns on the centrality of Aβ on AD onset and other hypotheses has been advanced in the years (Nunomura et al., 2006; Crowley, 2014; Cervellati et al., 2016). Noteworthy, in spite of the variety of proposals in this framework, inflammation, and oxidative stress always emerged as possible interconnected fundamental components of AD pathogenesis and pathophysiology (Zhao and Zhao, 2013; Cervellati et al., 2016).

One of the central principle of the proposed concept of “OxInflmmation” is that the dynamic and self-perpetuating connection between the two short-circuits (biochemical and immune) is not locally (CNS) confined but reverberates at systemic level. AD properly fits this definition, since systemic manifestations, including inflammation and oxidative stress (as well as diabetes and other metabolic dysfunctions) are not merely risk factors of the diseases, but coexist and drive (and are driven by) neurodegeneration (Leuner et al., 2012; Metti and Cauley, 2012; Cervellati et al., 2014a, 2016; Morris et al., 2014). Consistent with this scenario, peripheral inflammatory markers such as C-reactive protein (CRP), IL-6 and TNF-α, have been repeatedly, although not always, found to be cross-sectionally and longitudinally associated with an increased risk of AD (Metti and Cauley, 2012). These findings are not surprising since the hypothesis of a communication between the systemic immune system and the CNS is gaining wide acceptance (Minihane et al., 2015). According to this, it has been shown that a systemic and chronic presence of proinflammatory cytokines can lead to the brain's activation of the innate immune system, prelude of neuroinflammation, an important component of AD pathogenesis and physiopathology (Heneka et al., 2015). Microglia, brain resident macrophages, provide the most significant innate and adaptive immune responses and function as CNS “sensor” of alteration the peripheral immune homeostasis (Heneka et al., 2015). Notably, animal experiments have shown that in the aging CNS, microglia exhibit enhanced sensitivity to inflammatory stimuli, so highly susceptible of further stimulation by inflammatory mediators (in primis, IL-6, TNF-α and IL-1β) that can easily cross the blood brain barrier (BBB) (Banks et al., 1995; Metti and Cauley, 2012; Perry and Teeling, 2013). In turn, this detrimental cross-talk entails the production of neurotoxic cytokines, chemokines, prostanoids as well as reactive oxygen species, which has been suggested to contribute to the formation and/or toxicity of Aβ and neurofibrillary tangles (Patel et al., 2005; Heneka et al., 2015).

NOX (mostly NOX2), localized not only in microglia but also in astrocytes and neurons, plays a central role in the aforementioned detrimental processes occurring since the early stage of AD. It has been shown that exposition of Aβ to microglia results in respiratory burst due to NOX activation and release of superoxide anion, and pro-inflammatory cytokines and chemokines (Mander and Brown, 2005; Wilkinson and Landreth, 2006). In turn, the soluble mediators derived from NOXs of these brain resident cells but also, as discussed in the previous paragraph, of peripheral immune cells, can further activate NOX and exacerbate OxInflammation. Furthermore, the cytokines can also stimulate inducible nitric oxide synthase (iNOS) in microglia and astroglia, producing high concentrations of nitric oxide (Mander and Brown, 2005). As clearly shown by Mender et al. (Mander and Brown, 2005), when iNOS and microglial NOX are simultaneously activated can lead to neurons death. Indeed, as mentioned earlier, nitric oxide becomes (neuro) cytotoxic only in presence of high concentration of superoxide anion, leading to the formation of peroxynitrite. This RNS, in turn, can mediate the post-translational modification of Aβ peptide and the nitration of these peptides has been shown to increase the propensity of Aβ to aggregate and to initiate plaque formation (Heneka et al., 2015).

Oxidative stress due to NOX over-activation or mitochondrial dysfunction has been also envisaged to be, not a mere effect, but the primary trigger of Aβ and NFT formation in AD brain (Moreira et al., 2010; Cai et al., 2011; Cervellati et al., 2016). The generation of Aβ occurs through two sequential cleavages of amyloid precursor protein (APP), elicited through β-secretase and γ-secretase. Experiments on animal models showed that oxidative stress significantly increases the catalytic activity of these two enzymes, which in turn augments Aβ production (Praticò et al., 2001). The deposition of Aβ in the neuronal tissue could reflect a compromised blood–brain barrier (BBB) and oxidative stress could contribute to damaging BBB either directly or through the activation of metalloproteinases (Cai et al., 2011; Aliev et al., 2014; Cervellati et al., 2016). As a consequence of the loss in BBB physical integrity, influx of Aβ from cerebrospinal fluid (CSF) and noxious substances (such as oxidants and pro-inflammatory agents) form systemic circulation, may increase and exacerbate AD pathological alterations.

In a similar fashion, oxidative stress can be both a downstream and upstream factor for NFT formation (Chauhan and Chauhan, 2006). These oligomers and toxic filaments are the result of aberrant hyperphosphorylation of microtubule-associated protein tau, which, in turn, is due to the dysregulated activities of kinases and phosphatases (Chauhan and Chauhan, 2006). It has been reported that abnormal polymerization of tau might be promoted by lipid peroxidation process, which is indirectly catalyzed by excess of iron present in intraneuronal NFT (Cristóvão et al., 2016). Accordingly, treatment of primary rat cortical neuron cultures with iron plus hydrogen peroxide enhanced tau hyperphosphorylation (Lovell et al., 2004). This aberrant process was also shown to be primed by antioxidant deficiency in AD transgenic mice, most likely because oxidative stress can activate one of the kinases (p38 MAPK) putatively involved in tau hyperphosphorylation (Giraldo et al., 2014). As observed for Aβ, also the neurotoxic effect of tau might be at least partially mediated by oxidative damage. Consistently, in a drosophila model of human tauopathies, the induced decrease of antioxidant enzymes worsened tau-related neurodegeneration, while upregulation attenuated this detrimental process (Dias-Santagata et al., 2007). The link between tau and oxidative stress was further demonstrated by studies on transgenic mice overexpressing tau gene and developed NFT; extensive mitochondrial dysfunctions associated with increased production of ROS and protein carbonyl levels were found to chiefly characterize the brain of these animals (Dumont et al., 2011).

To close the here proposed OxInflammation loop, it has been hypothesized that also neuro-inflammation could participate in the formation of NFT. The most compelling proof in support of this link was obtained by Kitazawa et al, who showed that administration of a known inducer of brain inflammation increased tau hyperphosphorylation, but not Aβ accumulation, in AD transgenic mice that develop both pathological hallmarks in an age-dependent manner in disease-relevant brain regions (Kitazawa, 2005). It has been also shown that tau dysfunction (characterizing animal models of tauopathies) is associated with a dysregulation of cytokine secretion by microglia, eventually enhancing tau p38 MAPK-dependent hyperphosphorylation (Bolós et al., 2017).

## Rett syndrome

The concept of OxInflammation has been firstly proposed in the pathogenic processes of RTT (Pecorelli et al., 2016b; Valacchi et al., 2017). This genetic disease is characterized by a parallel alteration of systemic inflammation and severely compromised redox balance, with the latter aberration repeatedly found in several models including mouse brain, non-neuronal tissues (e.g., fibroblasts), CSF and serum/plasma (Sierra et al., 2001; Pecorelli et al., 2011; De Felice et al., 2014; Cervellati et al., 2015b; Hayek et al., 2017).

Redox homeostasis dysregulation in RTT appeared to be the result of concentric alterations in antioxidant defensive mechanisms, mitochondria dysfunction and abnormal constitutive activation of NOX2 (Cervellati et al., 2015b; Pecorelli et al., 2016b). This last phenomenon has been explained as possible result of the chronic stimuli of marked and chronic immune dysregulation characterizing the typical RTT phenotype. A mechanism leading to self-perpetuating the inflammation-oxidative stress detrimental cycle in RTT might also be the chronic intermittent hypoxia (De Felice et al., 2009). This phenomenon is associated with some typical clinical manifestations of the genetic disorder, such as central apneas, significant obstructive apneas and hyperventilation (Hagberg, 2002). It is well-recognized that hypoxia can, in cooperation with oxidative stress, induce pro-inflammatory cytokines production via NF-κB, AP-1 and HIF-1-α activation (Cervellati et al., 2014b; Pecorelli et al., 2016b).

The most direct evidence in support of a role of oxidative stress in RTT development stem from the multiple findings of higher levels of lipoperoxidation byproducts, in particular 4-hydroxynonenal (4HNE), in peripheral cells and plasma of affected individuals compared to controls (Signorini et al., 2013). Under redox imbalance, 4HNE can indiscriminately react with proteins and nucleic acids, leading to cellular and tissue damage (Uchida, 2003). In particular, the formation of 4HNE-protein adducts (4HNE-PA), as a consequence of the covalent link between 4HNE and proteins, can irreversibly affect their structure/function, as reported in several diseases (Valacchi et al., 2017). In addition, the generation of these protein adducts can induce humoral immune responses (Kurien et al., 2006). The increase in 4HNE-PA levels leads, when it is accompanied by a unequate proteosome degrading activity, to intracellular and extracellular deposition of self-aggregating misfolded proteins. As shown by Kurien et al., this aberrant process results in the generation of neo-antigens that, once recognized by different immune receptors, are able to trig both innate and adaptive immune responses (Kurien et al., 2006). The impairment in proteosome machinery, and likely autophagy, observed in RTT cells, represents the ideal scenario for the accumulation of these immunogenic misfolded proteins and may contribute to the release of inflammatory mediators, which in turn contribute to the disease progression and severity (Cervellati et al., 2015b; Pecorelli et al., 2016c; Valacchi et al., 2017).

## Oxinflammation as fruitful source of pathological biomarkers

An ideal biomarker should be non-invasive (blood and urine are the most accessible specimens), enabling broader clinical access or eventually efficient population screening and reflect disease pathophysiology and be informative of the disease process, even in the early phase (Dalle-Donne et al., 2006). OxInflammation is a potential fruitful source of biomarkers that fit the above definition. This statement is true mainly for the most clinically and epidemiologically validated markers of (even subclinical) systemic inflammation such as Hs-CRP, IL6, IL8, IL1, and TNFα. In particular, Hs-CRP, although gap in knowledge of its function still remains, is the marker of choice for the evaluation of systemic inflammation and the most assessed in clinical studies, due to the high sensitivity/specificity (Danesh et al., 2000). The assessment of this marker allows the prompt and accurate quantification of the risk of most of the diseases now ascribed as inflammatory based, such as CVD, type II diabetes, metabolic syndrome etc. (Danesh et al., 2000; Pradhana et al., 2001; Reuben et al., 2002; Ansar and Ghosh, 2013). Besides, as mentioned in the previous paragraphs, elevated levels of hs-CRP, and its principal downstream inducers, including IL6, has been repeatedly found to be cross-sectionally associated with classically described CNS disorders such AD, vascular dementia, and RTT (Zuliani et al., 2007; Koyama et al., 2013; Cortelazzo et al., 2014).

The proposed concept of OxInflammation is further supported by the wealth of epidemiological/clinical evidence showing intercorrelation between peripheral (serum/plasma) markers of inflammation and those of oxidative stress (Pou et al., 2007; Il'yasova et al., 2008; Ouyang et al., 2009; Paltoglou et al., 2017).

In contrast with the aforementioned inflammation markers, which are measured by standardized and high-throughput methods, no validated peripheral index of oxidative stress is still available (Frijhoff et al., 2015; Cervellati and Bergamini, 2016). Indeed, despite years of intense research effort, there is a lack of consensus regarding the validation, standardization, and reproducibility of methods for the measurement (Dalle-Donne et al., 2006; Frijhoff et al., 2015). The biomarker discovery process in the oxidative stress field has been hindered by several methodological challenges, in particular the great complexity of the direct measurement of ROS/RNS in biological systems (Murphy et al., 2011). A number of analytical approaches can be used, such as electron spin resonance and mass spectrometry (MS) techniques, but none of them with application in clinical practice. As consequence of this intrinsic limitation, the most suitable approach is to assess these reactive species by evaluating the levels of their fingerprints, i.e., by-products of oxidatively damaged biomolecules (Frijhoff et al., 2015). Even for these more chemically stable molecules, problems regarding analytical specificity/sensitivity and other technical challenges still make difficult to translate these into clinical use. In the following paragraph (and Table 1), we briefly described the most important oxidative stress biomarkers, more details have been comprehensively described in recent reviews (Dalle-Donne et al., 2006; Frijhoff et al., 2015).

**Table 1:** Main biomarkers of oxidative stress-induced damage.

**Damaged biomolecules**	**Markers (common abbreviation)**	**Biological sample**	**Methods**	**Distinctive features and sources**
**LIPIDS (POLYUNSATURATED FATTY ACIDS)**				
	F_2_-isoprostanes (F_2_-iso)	urine/serum/plasma/cells/ breath/CSF	GC-MS/MS LC-MS/MS ELISA	•[-] Specifically derived from oxidation of arachidonic acid •[-] Widely regarded as the best marker of lipid-peroxidation (Dalle-Donne et al., 2006)
	Malondialdehyde (MDA)	serum/plasma/urine/cells	GC-MS/MS LC-MS/MS HPLC-FD Spectrophotometric assay Spectrofluorimetric assay	•[-] Physiologic ketoaldehyde produced by lipid- peroxidation •[-]The most assessed marker of lipid-peroxidation •[-] Spectrophotometric/spectrofluorimetric assays lack of specificity and sensitivity •[-] Can easily form stable protein adducts (Dalle-Donne et al., 2006)
	4-hydroxynonenal (4-HNE)	serum/plasma/cells	GC/MS ELISA	•[-] Aldehyde produced by lipid-peroxidation •[-] Can easily form stable protein adducts (Valacchi et al., 2017)
	Lipid hydroperoxides (LOOH)	serum/plasma	GC/MS Spectrophotometric assay Spectrofluorimetric assay	•[-] Relatively stable byproducts of lipid peroxidation •[-] Specificity/sensitivity problem of spectrophotometric/spectrofluorimetric methods
	2-propenal (acrolein)	serum/plasma/cells	LC–MS–MS LC/GC–MS Immunoblot ELISA	•[-] The most abundant aldehydes produced by lipid-peroxidation •[-] Reacts with DNA, phospholipids and protein (Tully et al., 2014)
	Oxidized low density lipoprotein (oxLDL)	serum/plasma	HPLC Spectrophotometric assay ELISA	•[-] Derived from oxidation of lipid component of LDL •[-] Well-established biomarker of cardiovascular disease risk •[-] The validity of oxLDL a marker of oxidative stress has been questioned (Frijhoff et al., 2015)
**PROTEINS**				
	Protein carbonyls	serum/plasma/CSF/cells	HPLC ELISA Immunoblot Spectrophotometric assays	•[-] Aldehydes and ketones produced from nonspecific oxidation of protein side chains •[-] Spectrophotometric and ELISA, although very unspecific, are the most frequently used assay methods (Dalle-Donne et al., 2006)
	Advanced Oxidation protein products (AOPP)	serum/plasma/urine	Spectrophotometric assays	•[-] Class of dityrosine-containing protein products •[-] Available high-throughput methods •[-] Analytical specificity problem (Cervellati et al., 2016)
	Nitrotyrosine (Tyr-NO_2_)	serum/plasma /urine	GC-MS/MS HPLC-MS/MS HPLC-ED ELISA	•[-] Stable byproduct of oxidation mediated peroxynitrite anion and nitrogen dioxide •[-] Specificity/sensitivity problem of immunological methods (Dalle-Donne et al., 2006)
**NUCLEIC ACIDS**				
	8-oxo-7,8-dihydro-2′-deoxyguanosine (8-OHdG)	urine/serum/plasma/CSF/cells	GC-MS/MS HPLC-MS/MS ELISA	•[-] Major product of oxidative DNA damage (Goto et al., 2008)
	8-hydroxyguanosine (8-OHG)	urine/serum/CSF/cells	HPLC-MS/MS HPLC ELISA	•[-] Major product of oxidative RNA damage (Henriksen et al., 2009)
**CARBOHYDRATES**				
	Advanced glycationend products (AGEs)	urine/plasma/serum/CSF/cells	LC-MS/MS HPLC ELISA Spectrophotometric assays	•[-] Byproducts of nonenzymatic reaction of reducing sugars with amino groups of lipids, DNA, and proteins •[-] Analytical methods are limited by high heterogeneity of AGEs (Frijhoff et al., 2015)

*CSF, cerebrospinal fluid; ELISA, enzyme linked immunosorbent assay; GC, gas chromatography; MS, mass spectrometry; MS/MS, tandem mass spectrometry; LC, liquid chromatography; HPLC, high pressure liquid chromatography; FD, fluorimetric detection; ED, electrochemical detection*.

By-products derived by peroxidation of polyunsaturated fatty acids (PUFAs) are the most assessed in epidemiological studies. Among these markers are “primary” products such as hydroperoxides (LOOH), or “secondary” products such as malondialdehyde (MDA), 4-hydroxynonenal (4-HNE), acrolein, and F_2_-isoprostanes (IsoPs). The reliability of these markers chiefly depends on the assay used to measure them. An emblematic example in this frame is MDA. The concertation of aldehyde can be measured in various biological specimens by using a fast and easy spectrophometric assay known as thiobarbituric acid reacting substances (TBARS); this method is one of the most commonly used in oxidative stress quantification, even if it is highly unspecific (Frijhoff et al., 2015). For an accurate detection of MDA serve more labor- and time-consuming methods such as high pressure liquid chromatography (HPLC) plus fluorimetric detection and gas chromatography (GC)—tandem MS (MS/MS) (Dalle-Donne et al., 2006). Similarly, the most reliable marker of lipid peroxidation, F_2_-isoprostane, by-products of free-radical mediated oxidation of arachidonic acid, is properly measured but GC-MS/MS and LC-MS, while the widespread commercial ELISAs are poorly accurate (Frijhoff et al., 2015; Cervellati and Bergamini, 2016).

Other widely used marker of oxidative stress are the byproducts generated by oxidative modification of proteins, such as carbonyl-groups 3-nitrotyrosine (formed by the reaction between the amino acid and peroxynitrite or NO), advanced oxidation protein products (AOPP), and those derived by modification of DNA, 8-oxo-7, 8-dihydro-2′-deoxyguanosine (8-OHdG), and RNA, 8-hydroxyguanosine (8-OHG), and carbohydrates (advanced glycation products, AGEs) (Dalle-Donne et al., 2006; Frijhoff et al., 2015).

As nicely reviewed by Frijhoff et al “additional value of oxidative stress biomarkers may come from being indicators of a disease mechanism common to several pathologies rather than diagnostic for a specific disease” (Frijhoff et al., 2015). The combination of biomarker of oxidative stress with those of inflammation may aid in risk stratification of several diseases, predicting and monitoring clinical progression in affected patients as well as in intercepting patient group that benefit from specific treatments.

## Concluding remarks

**Scheme 1:**
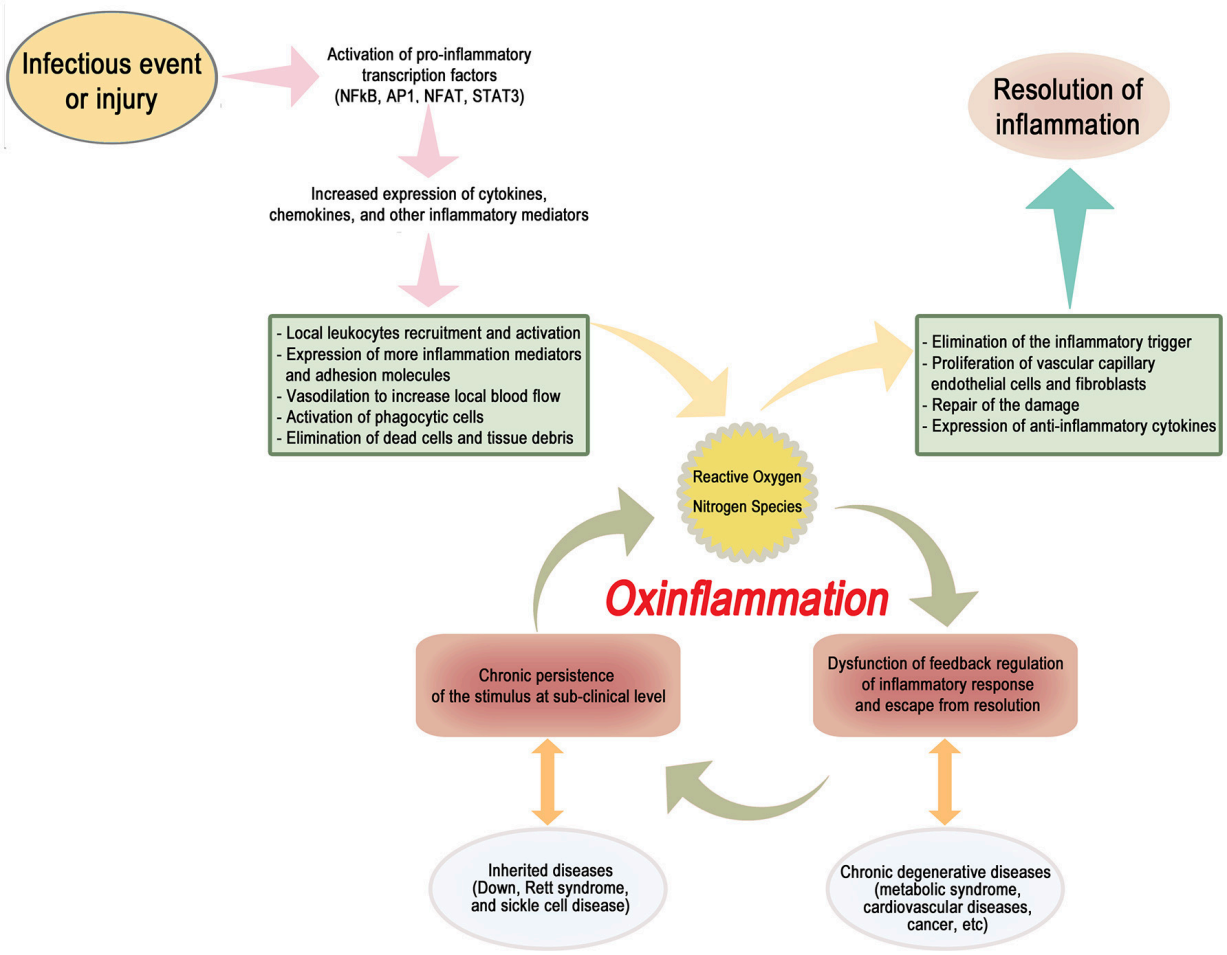
A dysfunction of a proper negative feedback regulation of the inflammatory response may result in the stabilization of a low-grade chronic activation of a vicious circle, characterized by a constant generation of a pro-oxidant environment, triggering in turn a sub-clinical systemic pro-inflammatory status. This condition, to be defined as “OxInflammation,” can induce a derangement from adaptive homeostatic capacities of the organism and eventually lead to overt pathological metabolic dysfunctions such as type II diabetes and related degenerative disease. OxInflammation plays an important role both in determining the clinical outcomes associated to several “genetic diseases,” such as Down syndrome, Rett Syndrome and sickle cells disease and also to other complex diseases determined by complex gene/environment interaction, such as metabolic syndrome and related degenerative complications, including cardiovascular diseases and cancer.

Here, we propose the term “OxInflammation” to describe a pre-pathological condition characterized by the well-documented chronic and systemic oxidative stress associated, within a vicious circle, to a mild-subclinical chronic inflammation (Scheme 1). The occurrence of a long-term sustained oxidative stress contributes to generate a permanent loss of the capacity to react by an adaptive homeostatic response, stabilizing and reinforcing a chronic induction of a pro-inflammatory status.

In other words, an initial stimulus, due to sporadic environmental events or originating from specific genetic features, triggers an inflammatory response. This response, if not properly quenched and terminated by appropriate negative feedback signals, results in a mild, low grade long term inflammation that is further propelled and stabilized by the sustained continuous production of pro-oxidant, electrophilic species. Electrophiles, such as reactive aldehydes (4-HNE), MDA, and F_2_-isoprostanes, have a role as inducers of inflammatory cells infiltration and activation, but are also able to directly react with DNA to form exocyclic DNA adducts, which have been detected in a variety of inflammatory diseases (Uchida, 2003). Finally, MDA and 4-HNE can react with protein thanks to the close interaction between lipids and proteins resulting in the synthesis of neo-antigens that in turn can further initiate and exacerbate systemic immune reactions, activation of stellate cells in several organs including brain, liver, pancreas and bones and neutrophil chemotaxis.

Therefore, a “localized” original inflammatory response results in the systemic dysfunction of the adaptive control of redox status, generating in turn a dysfunction of antioxidant control involving several organs.

This condition, characterized by a derangement from the threshold of adaptive redox homeostasis predisposes the organism to a continuous damaging effect of oxidative stress. This oxidative damage primes in turn a continuous sub-clinical pro-inflammatory response that is a common feature for several diseases, at the pre-clinical and clinical levels, and that can have a role in the developing of the pathological conditions.

## Author contributions

All authors listed have made a substantial, direct and intellectual contribution to the work, and approved it for publication.

### Conflict of interest statement

The authors declare that the research was conducted in the absence of any commercial or financial relationships that could be construed as a potential conflict of interest.
